# Cannabinoids: Well-Suited Candidates for the Treatment of Perinatal Brain Injury

**DOI:** 10.3390/brainsci3031043

**Published:** 2013-07-09

**Authors:** David Fernández-López, Ignacio Lizasoain, Maria Ángeles Moro, José Martínez-Orgado

**Affiliations:** 1 Neonatal Brain Disorders Center, Department of Neurology, University of California San Francisco, San Francisco, 94158 CA, USA; 2 Neurovascular Research Unit, Department of Pharmacology, School of Medicine, Complutense University of Madrid, Madrid 28040, Spain; E-Mails: ignacio.lizasoain@med.ucm.es (I.L.); neurona@med.ucm.es (M.A.M.); 3 Department of Neonatology-Pediatrics, University Hospital Puerta de Hierro Majadahonda, Madrid 28222, Spain; E-Mail: Jose.martinezo@salud.madrid.org

**Keywords:** cannabinoids, neonatal, hypoxia-ischemia, stroke, WIN55212-2, cannabidiol

## Abstract

Perinatal brain injury can be induced by a number of different damaging events occurring during or shortly after birth, including neonatal asphyxia, neonatal hypoxia-ischemia and stroke-induced focal ischemia. Typical manifestations of these conditions are the presence of glutamate excitoxicity, neuroinflammation and oxidative stress, the combination of which can potentially result in apoptotic-necrotic cell death, generation of brain lesions and long-lasting functional impairment. In spite of the high incidence of perinatal brain injury, the number of clinical interventions available for the treatment of the affected newborn babies is extremely limited. Hence, there is a dramatic need to develop new effective therapies aimed to prevent acute brain damage and enhance the endogenous mechanisms of long-term brain repair. The endocannabinoid system is an endogenous neuromodulatory system involved in the control of multiple central and peripheral functions. An early responder to neuronal injury, the endocannabinoid system has been described as an endogenous neuroprotective system that once activated can prevent glutamate excitotoxicity, intracellular calcium accumulation, activation of cell death pathways, microglia activation, neurovascular reactivity and infiltration of circulating leukocytes across the blood-brain barrier. The modulation of the endocannabinoid system has proven to be an effective neuroprotective strategy to prevent and reduce neonatal brain injury in different animal models and species. Also, the beneficial role of the endocannabinoid system on the control of the endogenous repairing responses (neurogenesis and white matter restoration) to neonatal brain injury has been described in independent studies. This review addresses the particular effects of several drugs that modulate the activity of the endocannabinoid system on the progression of different manifestations of perinatal brain injury during both the acute and chronic recovery phases using rodent and non-rodent animal models, and will provide a complete description of the known mechanisms that mediate such effects.

## 1. Introduction

Brain injury during the perinatal period may occur prior, during or shortly after birth, and can be triggered by a number of diverse conditions ranging from hypoxia-ischemia to brain trauma, metabolic diseases, genetic malformations and ischemic stroke. In this review we focus on the therapeutic potential of drugs that modify the activity of the endocannabinoid system (cannabinoids) for the treatment of newborn babies undergoing neonatal encephalopathy secondary to hypoxia-ischemia or perinatal asphyxia and neonatal arterial ischemic stroke. We present and discuss data obtained from *ex vivo* and *in vivo* rodent and non-rodent animal models in which cannabinoids showed beneficial effects during the acute, sub-acute and chronic phases of neonatal encephalopathy. Also, we address the specific molecular and cellular mechanisms that mediate some of the protective effects of cannabinoid administration characterized in those studies. 

## 2. Neonatal Hypoxic-Ischemic Encephalopathy (NHIE)

With an estimated incidence of 1 to 6 in 1000 live term newborns, hypoxic-ischemic encephalopathy is the most frequent type of acquired neonatal brain injury [1]. The incidence is even higher in pre-term newborns, affecting approximately a 60% of the babies [2,3]. The manifestation of brain injury differs depending on the maturational status of the brain at the time of the insult. In pre-term babies white matter injury is predominant due to the presence of a relatively abundant developing oligodendrocyte progenitor cell population that is selectively vulnerable to excitotoxicity and neuroinflammation induced by hypoxia-ischemia [4,5,6]. In contrast, in term babies, in which the maturation of the white matter is more advanced and the presence of oligodendrocyte progenitors is more reduced, neuronal degeneration in grey matter structures (e.g., basal ganglia and thalamic nuclei) is the most commonly observed manifestation of injury [7,8]. 

The pathophysiology of hypoxic-ischemic brain injury primarily involves a “triad” of events comprised by glutamate excitotoxicity, neuroinflammation and oxidative stress [9], the combination of which ultimately induces the activation of apoptotic and necrotic cell death pathways, resulting in a “continuum” of cell death states [10,11,12] and in the generation of permanent brain lesions accompanied by prolonged microgliosis and astrocyte reactivity [13]. Shortly after acute brain injury and during the initial stages of the chronic recovery phase the neonatal brain initiates a series of endogenous responses aimed to promote intrinsic mechanisms of neural repair in the affected regions. These responses include the induction of neural stem cell proliferation in the subventricular zone (SVZ) with the subsequent generation of new progenitors of all three neural lineages [14,15,16]. Some of these progenitors are able to survive and migrate towards the boundaries of the injured brain regions, where they can differentiate into mature oligodendrocytes, astrocytes and even neurons [16,17]. However, the extent and significance of this neurogenic response is unclear, since only a very small proportion of neuronal progenitors survive and differentiate into fully mature and functionally integrated neurons. Also, while in the hypoxic-ischemic pre-term brain numerous oligodendrocyte progenitors are generated *de novo* in response to injury, their maturation is abnormal and stays arrested in a pre-oligodendrocyte state [18]. 

Multiple therapeutic strategies for the amelioration of neonatal hypoxic-ischemic brain injury have been investigated at the experimental level [19]. Those studies have been aimed to test the capacity of different potential therapies for the prevention of brain injury and, in some cases, for the promotion of brain repair during the chronic recovery phase. So far, hypothermia (applied as head or whole-body cooling) is the only clinical intervention that has shown some effectiveness in reducing brain damage in hypoxic-ischemic newborn babies [20,21]. However, hypothermia does not prevent injury in all cases and, although newborns with moderate to severe hypoxic-ischemic encephalopathy also benefit from mild hypothermia, the treatment does not always lead to complete functional recovery [22]. Therefore, the use of effective adjuvant drugs that could be administered in conjunction with hypothermia may result in a higher therapeutic effectiveness in those cases in which hypothermia results in partial or no improvement. Experimental studies have been focused on drugs with known capacity to inhibit glutamate excitotoxicity (e.g., magnesium sulfate, topiramate, xenon) [23,24,25,26], reduce oxidative stress (e.g., *N*-acetylcysteine, allopurinol) [27,28,29], modulate the neuroinflammatory response (e.g., minocycline) [30] or affect multiple endogenous processes involved in brain injury and recovery (e.g., pleiotropic drugs like erythropoietin) [31,32,33]. Cannabinoids fall into the latter group since, as it is further described below, they can modulate a wide range of cellular pathways and pathological mechanisms that participate in the generation of perinatal brain injury [34]. Cannabinoids are also known to control the cell cycle and the proliferation, survival and differentiation of neural stem cells [35,36], capabilities that target them as interesting experimental drugs for the enhancement of endogenous brain repair responses to perinatal brain injury. 

## 3. Neonatal Arterial Ischemic Stroke

Recent clinical data have revealed that the incidence of neonatal stroke is higher than it was initially presumed. Neonatal arterial ischemic stroke occurs in 1 in 2300–4000 full term births [37,38,39], and in approximately 0.7% of pre-term births [40]. Although neonatal stroke presents with relatively reduced mortality [41], adverse long-term *sequelae* are commonly detected during the early childhood. Around one third of the newborns affected by stroke develop unilateral cerebral palsy, and learning and cognitive deficits are also frequent [37,42]. The risk factors that favor the occurrence of stroke in newborn babies can be maternal (e.g., history of infertility, gestational diabetes, pre-eclampsia), antepartum (e.g., oligohydramnios), intrapartum (e.g., chorioamnionitis, placental thrombosis) or postnatal (e.g., infection, congenital heart diseases, prothrombotic states) [43]. Many of the pathophysiological mechanisms that lead to brain injury are common for hypoxic-ischemic encephalopathy and neonatal stroke, although the final patterns of injury can differ significantly in the case of neonatal stroke depending on the extension and selective vulnerability of the areas irrigated by the occluded artery, as well as on the duration of the artery occlusion. 

As it happens in the case of neonatal hypoxia-ischemia, therapeutic interventions available for the treatment of the babies affected by neonatal stroke are very limited. Thus, there is a dramatic need to develop novel effective therapies for the prevention and correct management of brain injury induced by neonatal stroke, as well as for promoting functional recovery during the chronic stage of the disease. 

## 4. The Endocannabinoid System

**Figure 1:**
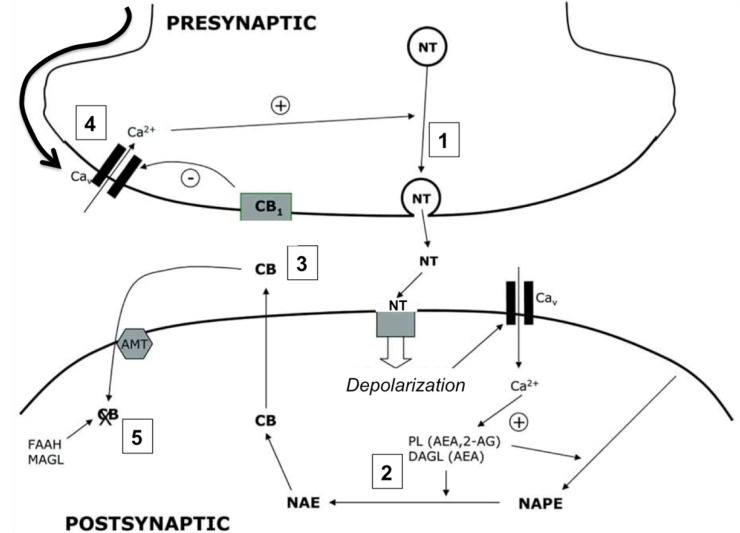
Cannabinoids as mediators of neuronal retrograde signaling. The presence of cannabinoid receptors (CBRs) on presynaptic neurons modulates the release of neurotransmitter to the synapsis. The action potential in the presynaptic neuron causes the fusion of neurotransmitter vesicles with the plasma membrane (1). The binding of the neurotransmitter to its postsynaptic receptors induces the depolarization of the postsynaptic membrane and the accumulation of Ca^2+^ in the cytoplasm, inducing the activation of calcium-dependent enzymes in charge of the biosynthesis of endocannabinoids (PL, DAGL) (2). Cannabinoids produced *de novo* diffuse through the postsynaptic membrane, binding to the presynaptic CBRs (3). The activation of CBRs promotes the hyperpolarization of the presynaptic membrane (4) and modulates the release of neurotransmitter, regulating synaptic transmission. Endocannabinoids are internalized by a selective transporter (AMT) and degraded by specific enzymes (FAAH, MAGL) (5).

The endocannabinoid system is a neuromodulatory system comprised by endogenous ligands or endocannabinoids (being *N*-arachidonoylethanolamine, anandamide or AEA, and 2-arachidonoylglycerol, 2-AG, the most abundant), specific and non-specific receptors for both endogenous and exogenous ligands and enzymes that degrade endocannabinoids in the cytosol [44]. It has been proposed that the uptake of endocannabinoids from the extracellular space occurs by facilitated diffusion mediated by a selective transporter, although experimental evidences for the existence of such molecule remain controversial [45]. The specific cannabinoid receptors (CBRs) are expressed in numerous cell types in the body and modulate a plethora of biological functions. In the central nervous system (CNS) the endocannabinoid system participates, among other functions, in the control of motor coordination, memory and learning, body temperature, appetite and pain [46,47]. Most neural functions controlled by endocannabinoid signaling depend on the neuronal cannabinoid receptor type 1 (CBR1). This receptor is the primary mediator of the inhibition of neurotransmission by retrograde signaling mediated by endocannabinoids [48], a neuromodulatory mechanism of neuronal depolarization and neurotransmitter release that is described in more detail in Figure 1. 

### 4.1. Cannabinergic Ligands

Apart from the two main endocannabinoids, anandamide and 2-AG, other endogenous compounds including noladin eter, *N*-arachidonoyldopamine and virhodamine also show high affinity for CBRs and are able to activate them [44,49]. The exogenous cannabinergic ligands are also numerous and diverse, and can be classified in into three different groups according to their origin and/or chemical structure [49]: (1) classical cannabinoids, that includes all natural active principles of the plant *Cannabis sativa* (e.g., delta-9-tetrahydrocannabinol (THC) and cannabidiol) as well as their synthetically modified derivatives; (2) non-classical cannabinoids, a family of synthetic bi- or tricyclic compounds, being CP55,940 the most representative; and (3) aminoalkylindoles, where WIN55212-2 is the most commonly used in experimental research. Both endogenous and exogenous cannabinoids have different relative affinities for the two main CBRs (CBR1 and CBR2), which explain the broad spectrum of effects triggered by their different ligands [50].

NT: neurotransmitter. Ca^2+^: Calcium. NAPE: *N*-acyl-phosphatidylethanolamine. NAE: *N*-acyl-ethanolamine. PL: phospholipase. DAGL: diacylglycerol lipase. CB: endocannabinoid. AMT: anandamide membrane transporter. FAAH: fatty acid amide hydrolase. MAGL: monoacylglycerol lipase.

### 4.2. Cannabinoid Receptors (CBRs)

**Figure 2:**
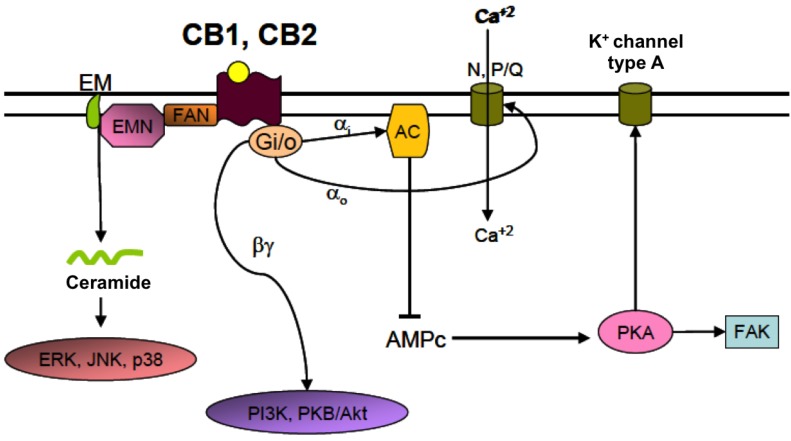
Main signaling pathways activated by cannabinoid receptors. The canonical signaling pathway initiated by the binding of a cannabinoid to CBRs involves the coupling of the receptor to G_i/0_ proteins. α_i_ subunits can inhibit the activity of adenylyl cyclase (AC) and the synthesis of cAMP. This results in a decreased activation of PKA and an increased activation of potassium channels type A, which leads to membrane hyperpolarization. α_0_ subunits can in turn inhibit voltage dependent Ca^2+^ channels contributing to the inhibition of membrane depolarization. βγ subunits interact with other intracellular pathways related to PI3K or PKB/Akt. CBRs are also coupled to neutral sphingomyelinase (EMN), an enzyme that mediates the generation of ceramide from sphingomyelin (EM) in the plasma membrane. Ceramide acts as an intracellular signaling molecule than can activate several transcription factors including ERK, JNK and p38, and is involved, among other functions, on the control of cell fate and survival. AC: adenylyl cyclase; FAN: factor associated with neutral sphingomyelinase activation; N, P/Q: voltage-dependent calcium channels type N, P/Q; PKA: protein kinase A; PKB/Akt: protein kinase B; ERK: extracellular signal-regulated kinase; JNK: c-Jun *N*-terminal kinase; FAK: focal adhesion kinase; PI3K: phosphoinositide-3 kinase.

Two main specific receptors for endocannabinoids were cloned and characterized in the early 1990s: CB1, which in the central nervous system (CNS) is located primarily in neurons, and CB2, expressed in activated microglia and astrocytes and other immune cells that may infiltrate in the CNS under pathological conditions [51,52,53]. Both are G protein-coupled receptors, although they have been shown to associate with other intracellular signaling molecules as well. The canonical signaling pathway for CBRs involves their coupling with G_i/__0_ and normally results in an overall inhibitory signal. Other pathways that can be activated by the binding of cannabinoids to CBRs involve the enzymes PI3 kinase, esphingomyelinase, and phospholipase C [54] (Figure 2). The final effect of a certain cannabinergic ligand on the biology of the cell will depend on the affinity of the ligand for the receptor, the cell type, the subcellular location of the receptors and the tissue context in which the cell is placed [55]. In postsynaptic neurons, endocannabinoids are produced on demand in response to membrane depolarization upon Ca^2+^-dependent activation of the enzymes in charge of their biosynthesis. Endocannabinoids diffuse across the plasma membrane and reach the CB1R receptors located in the presynaptic membrane, reducing its depolarization state and inhibiting neurotransmitter release [48]. Thus, through CB1R the endocannabinoid system modulates the intensity and duration of synaptic transmission. Similarly, in immune cells (including microglia) CB2R activation has been shown to mediate an inhibitory effect on activation, cell motility and secretion of inflammatory mediators [56,57]. The intrinsic capability of the endocannabinoid system of inhibiting synaptic transmission and immune responses has arisen the interest on cannabinoids as therapeutic molecules for the prevention and treatment of CNS pathologies with an important neuroinflammatory component [58], being several manifestations of perinatal brain injury included in this group. 

## 5. Mechanisms of Action of Cannabinoids in Experimental Models of Perinatal Brain Injury

Cannabinoids induce a number of cellular responses mediated by their binding to CBRs that have been proven beneficial for neuroprotection and neural repair following multiple experimental paradigms of brain injury. Cannabinoids modulate neuronal excitability and counteract glutamate excitotoxicity [59,60,61]. Moreover, through the activation of CB2R in immune cells cannabinoids can modulate the intensity and extension of neuroinflammatory responses to injury [57,62]. CBRs also play a role in the control of cell survival and fate [63,64], and have been shown to mediate neural stem cell proliferation [65,66] and differentiation [67,68] of neural progenitors, which is of great clinical interest for the enhancement of the endogenous restorative responses to neonatal brain injury. Other potentially neuroprotective effects of cannabinoids are the induction of hypothermia (mediated by CB1R) and the antioxidant capacity of some cannabinoids with specific chemical structures [69]. Finally, cannabinoids have been shown to decrease vascular reactivity and the expression of adhesion molecules in endothelial cells, thus reducing the rolling and adhesion of circulating leukocytes and preventing their infiltration into the inflamed brain [70,71]. All the mentioned mechanisms have a great potential to prevent brain damage and promote brain recovery following neonatal brain injury. The multiple actions of cannabinoids on different key mechanisms governing cell death, inflammatory responses and endogenous repair mechanisms awakened the interest on these compounds as potential drugs for the prevention and treatment of neonatal brain injury. 

### 5.1. Rodent Models of Neonatal Hypoxic-Ischemic Encephalopathy

Based on previous experimental data showing the particular inhibitory effects of cannabinoids on intracellular calcium accumulation [72,73], glutamate release from neurons [60], cytokine production in microglia [62] and expression of inducible nitric oxide synthase (iNOS) [74,75] (among other potentially neuroprotective effects), an initial study was designed to test the therapeutic potential of a synthetic CBR1 and CBR2 full agonist, WIN55,212 (WIN), on an *ex vivo* model of NHIE. In this study brain slices were obtained from neonatal (P7) rats and subjected to acute oxygen and glucose deprivation (OGD) for a period of 30 min [76]. OGD induced glutamate release to the incubation medium and caused cell death (measured both by LDH release and histological analysis) up to 2 h after replacement of the medium with oxygen and glucose [76,77]. The accumulation of the inflammatory cytokine tumor necrosis factor alpha (TNFα) was also increased in the incubation medium after OGD, and the protein expression of iNOS was induced in the brain slices [76,77]. Pre-incubation of the brain slices with WIN (50 mM) prevented glutamate release, TNFα accumulation and iNOS induction, resulting in a reduction of cell death induced by OGD [76]. The protective effect of WIN was dependent on the simultaneous activation of both CBR1 and CBR2, since the co-incubation of WIN with either the selective CBR1 antagonist rimonabant or the CB2R antagonist SR144528 completely reversed the protective effect of the cannabinoid in both cases [76]. Furthermore, the use of selective agonists for CBR1 (ACEA) or CBR2 (JWH-133), only led to partial protection when compared with WIN [75]. 

The neuroprotective capacity of WIN was further explored using an *in vivo* rat model of NHIE, the Rice-Vannucci model [78]. The administration of WIN (1 mg/kg) immediately after hypoxia-ischemia prevented the generation of cytotoxic and vasogenic edema (evaluated by MRI using a combination of DWI and T2WI from 24 h to 7 days after HI), and reduced the apparent diffusion coefficients (ADCs) in the injured brain regions at 7 days after HI [78]. These effects were concomitant to a better preservation of cell integrity evaluated by histology at the same time point. Again, the co-administration of WIN with either rimonabant or SR144528 completely reversed the protective effect of WIN, confirming the previous observation that the simultaneous activation of both CBR1 and CBR2 by WIN is required for protection [76,79]. 

Cannabinoids have also been proven to enhance mechanisms of neural recovery and repair following neonatal HI. The endocannabinoid system had been shown to modulate neural stem cell proliferation through CBR2 and to shift differentiation to the astroglial phenotype in detriment of the differentiation into the neuronal lineage through CB1R [65,66,67]. The stimulatory effect of CBR2 activation on neural stem cell proliferation relies on the PI3K/Akt/mTORC1 signaling pathway and the downstream inhibition of the cell-cycle regulatory protein p27Kip1 [80]. Interestingly, under excitotoxic conditions CBR1 promotes the differentiation of neural stem cells towards the neuronal lineage [68], indicating that the context in which CB1R is activated (healthy *vs.* injured brain) may condition the polarization of neural stem cells towards a specific cell lineage. Adult and neural stem cells can also promote the survival of pre-existing neurons by the release of anandamide and the prevention of glutamate-dependent excitatory currents and subsequent excitotoxicity [81]. 

Using the Rice-Vannucci model, WIN was administered in two daily doses of 1 mg/kg during the 7 days posterior to HI. The prolonged administration of WIN promoted neural stem cell proliferation in the SVZ at 7 days after injury, but not at 14 or 28 days, relating this effect to the period of administration of WIN [82]. Interestingly, the number of neuroblasts present in the injured caudate was increased at 14 days, but not at 28 days after injury. The decrease in the number of neuroblasts was not due to the differentiation of these cells into mature neurons as it could be expected, since at 28 days after HI very few newly-generated mature neurons were observed in the injured caudate. These data indicate that, while WIN promotes neural stem cell proliferation in the SVZ and the short-term generation and migration of neuroblasts to the adjacent injured caudate, the long-term survival of these cells is minimal once the cannabinoid treatment is withdrawn. The question of whether a more prolonged administration of WIN would favor the survival and differentiation of the newly-generated neuroblasts into functional, mature neurons is still open. 

One of the most interesting beneficial effects of WIN following neonatal HI is related to white matter recovery after injury. The modulation of oligodendrocyte differentiation by cannabinoids during the first postnatal days has been characterized in naïve animals. One study showed that the administration of the selective CB1R agonist ACEA during the early postnatal period (P1 to P14) increases the generation of oligodendrocyte progenitors (Olig2-expressing cells) in the SVZ [83]. In the same study, the administration of WIN promoted the myelination of the subcortical white matter, an effect that was abrogated by the administration of either rimonabant or SR144528 [83]. Further studies revealed that CB1R, CB2R and CB1R/CB2R agonists enhance oligodendrocyte differentiation by activating PI3K/Akt and mTOR signaling, illustrating some of the signaling pathways involved in the control of this cellular process [84]. Interestingly, the interference with constitutive 2-AG synthesis reduced oligodendrocyte differentiation, while the inhibition of the main 2-AG degrading enzyme had the opposite effect [85], implicating an intrinsic regulatory role of the endocannabinoid system on the control of oligodendrocyte maturation. Consistently with these data, the administration of WIN during the first 7 days after experimental HI led to increased number of NG2^+^ oligodendrocyte progenitors in the external capsule of the injured brain hemisphere by the end of the treatment [82]. Some of these newly-generated oligodendrocyte progenitors were able to survive and differentiate into mature, myelinating oligodendrocytes in the external capsule and in the injured caudate at 14 and 28 days after HI, which was reflected in an accelerated myelin restoration in the external capsule in the WIN-treated animals [82]. Together, these data strongly suggest that WIN promotes oligodendrocyte generation, survival and differentiation, contributing to a faster restoration of myelin in the white matter areas acutely affected by HI. However, the functional implications of these effects are yet to be determined, since no behavioral or functional tests were performed in these studies. 

The specific molecules and signaling pathways involved in the protective effect of cannabidiol (CBD), a natural cannabinoid with no psychoactive effects, were explored using an *ex vivo* rodent model of NHIE [86]. In this study brain slices were incubated with CBD (100 mM) and subjected to OGD 15 min after. Pre-incubation with CBD prevented acute LDH and glutamate accumulation in the incubation medium after OGD. Interestingly, caspase-9 concentration in the brain slices was also reduced by CBD, suggesting that both necrotic and apoptotic pathways are inhibited. The induction of the expression of several pro-inflammatory mediators (IL-6, TNFα, COX-2 and iNOS) was also reduced by incubation of brain slices with CBD prior to OGD. The more interesting piece of data from this study refers to the specific receptor mediating the protective effects of CBD. Selective antagonists of CB2R and the adenosine receptors A_1A_ and A_2A_ were used in combination with CBD in order to identify the molecular targets of this cannabinoid. CB2R antagonism reversed most effects of CBD, leading to increased cell death, glutamate accumulation and expression of inflammatory mediators. Interestingly, antagonism of A_2A_ adenosine receptor led to a similar reversion of CBD actions, while A_1A_ antagonism exerted a more discrete effect. The identification of A_2A_ as a receptor mediating the protective effects of CBD arose the question of whether CBD is acting on A_2A_ by a direct binding or by an indirect effect on the modulation of adenosine levels. Further experiments are required in order to address this question. Finally, the long-lasting protective effect of CBD was tested using the Rice-Vannucci model [87]. In this model CBD (1 mg/kg) induced a sustained reduction in excitotoxic damage, oxidative stress and inflammation up to 7 days after injury, addressing the three major pathophysiological mechanisms involved in the generation of permanent brain damage after HI. Moreover, long-lasting functional impairment was prevented by CBD administration, while no side effects of the drug were observed [86]. 

### 5.2. Non-Rodent Models of Neonatal Hypoxic-Ischemic Encephalopathy

The prospects of cannabinoids becoming effective and safe therapeutic drugs against neonatal hypoxia-ischemia increased considerably after several studies demonstrated the beneficial effects of CBD on multiple neurophysiological parameters commonly used for the bedside monitoring of asphyxiated newborns using a model of NHIE in newborn piglets. In one of those studies the intravenous administration of CBD (0.1 mg/kg) improved brain tissue oxygenation during the first 3 h after HI, and induced a partial recovery of EEG amplitude from 1 to 6 h [88]. Moreover, the increase in cerebral impedance (a parameter used to detect the presence of cerebral edema) induced by HI was more modest in the piglets that received CBD. Histological analysis of the brains 6 h after HI revealed that the presence of pyknotic and degenerating cells was reduced by CBD. The same study ruled out possible side effects of CBD on several respiratory and cardiovascular parameters, suggesting that CBD may be an effective and safe drug to use in hypoxic-ischemic newborns. Follow-up experiments using a similar experimental setting showed that the protective effects of the CBD are sustained beyond the acute phase of hypoxic-ischemic brain damage. Improvement on tissue oxygenation index and EEG amplitude was observed up to 72 h after HI when CBD was give in two doses at 15 and 240 min after HI [89]. The neurobehavioral analysis showed that the animals that were treated with CBD had a faster and more significant functional recovery compared to animals that received vehicle. The reduction in the number of TNFα^+^ cells was proposed as one of the possible protective mechanisms of action of CBD. 

Considering the promising results obtained using the CB1R and CB2R agonist WIN in rodent models of NHIE, this cannabinoid was also tested in a non-rodent animal model of perinatal asphyxia [90]. In this model, fetal lambs were induced brain injury by occlusion of the umbilical cord and reduction of blood flow to less than 50% for 60 min. A single, low dose of WIN (0.01 mg/mL) was injected intravenously immediately after delivery. The effect of WIN on the extent and modes of cell death, normal mitochondrial function and intracellular calcium accumulation were analyzed three hours after the induction of perinatal brain injury. WIN reduced the number of apoptotic cells (annexin5^+^/propidium iodide^−^), but did not affect the total number of necrotic cells (Annexin5^−^/propidium iodide^+^). Consistently, the number of viable cells (Annexin5^−^/propidium iodide^−^) was higher in lambs that received WIN. Mitochondrial function was also better preserved in WIN-treated animals, and calcium accumulation was lower in certain brain regions typically affected in this model, including the basal ganglia and the cerebellum. 

Altogether, these studies using both rodent and non-rodent animal models of NHIE strongly suggest that CBD may be a safe and effective drug to be used during the acute and sub-acute phases after NHIE, and is currently the best suited cannabinoid to be implemented for clinical use in babies affected by neonatal encephalopathy. 

### 5.3. Rodent Model of Neonatal Stroke

The beneficial effects of WIN on rodent and non-rodent models of NHIE strongly encouraged the study of the effects of this cannabinoid in experimental models of neonatal stroke. The interest of exploring potential drug therapies for the treatment of neonatal stroke is high since, as it has been mentioned before, stroke in newborn babies is a relatively frequent phenomenon for which no clearly effective therapies are yet available. Neonatal stroke was induced by middle cerebral artery occlusion after the insertion of a coated nylon monofilament through the internal carotid artery in P7 rats. Occlusion lasted for 90 min and was followed by reperfusion of the brain after removal of the monofilament [91]. Injection of WIN (1 mg/kg) twice daily during the first 72 h reduced infarct volume and accumulation of Iba1^+^ microglia/macrophages in both the core and periphery of the injury [92]. This effect was concomitant to a drastic up-regulation of CB2R and a reduction on Iba1 protein expression in the injured brain 24 h after neonatal stroke. In a previous study using the same animal model it was shown that monocyte infiltration in the neonatal ischemic brain is discrete during the first 24 h after reperfusion [93]. These observations suggest that WIN is acting on CB2R expressed in resident microglia. WIN also reduced Iba1^+^ cell proliferation at 72 h after injury, suggesting a possible mechanism explaining the reduced density of microglia/macrophages in these animals. However, further studies using either CB2R knock-out mice or selective CB2R antagonists need to be performed in order to confirm this hypothesis. In adult models of stroke and LPS-induced neuroinflammation, the protective effect of CB2R agonists has also been related to the modulation of inflammatory responses, including reduced microglia activation, expression of endothelial adhesion molecules and neutrophil recruitment [70,71,94]. Although the protective effect of WIN after neonatal stroke may also rely on other yet unexplored molecular and cellular mechanisms, the modulation of microglia/macrophage accumulation seems to be a key process contributing to the reduction of infarct size during the acute and sub-acute phases after injury. 

## 6. Final Remarks

Numerous experimental studies have proven that the modulation of the endocannabinoid system either by enhancement of the endogenous cannabinoid signaling or by administration of exogenous cannabinergic ligands has beneficial effects during the acute and recovery phases after perinatal brain injury. These studies covered different modes of neonatal brain injury including NHIE, perinatal asphyxia and neonatal stroke, and revealed the high potential of the endocannabinoid system as a novel therapeutic target for the prevention of permanent brain damage in newborns. The characterization of CBD, a non-psycoactive cannabinoid, as a safe and effective protective drug in non-rodent models of NHIE further encouraged the use of CBD as an adjuvant therapy for the acute treatment of the affected newborns. 
